# Efficacy and complication of endoscopic submucosal dissection for superficial esophageal carcinoma: a systematic review and meta-analysis

**DOI:** 10.1186/1749-8090-9-78

**Published:** 2014-05-07

**Authors:** Fenghao Sun, Ping Yuan, Tianxiang Chen, Jian Hu

**Affiliations:** 1 Department of Thoracic surgery, First Affiliated Hospital, Zhejiang University, Hangzhou, Zhejiang Province, China

**Keywords:** Superficial esophageal carcinoma, Endoscopic submucosal dissection (ESD), *en bloc*, R0 resection rate

## Abstract

**Aim:**

For patients with superficial esophageal carcinoma, ESD was one of treatment modalities to remove the lesion safely and effectively. We perform this meta-analysis to determine the efficacy and incidence of complication of ESD for patients with superficial esophageal carcinoma.

**Method:**

Articles were searched in MEDLINE (PubMed and Ovid), Cochrane Database of Systemic Reviews, Google scholar, and Web of Science. Two reviewers independently searched and extracted data. Meta-analysis of the efficacy of ESD was analyzed by calculating pooled *en bloc* and R0 resection rate. Incidence of complications such as perforation, stenosis and mediastinal emphysema was also calculated. Pooling was conducted using either fixed-effects model or random-effects model depending on the heterogeneity across studies.

**Results:**

21 studies (1152 patients and 1240 lesions) were included in this analysis. The pooled *en bloc* resection rate was 99% (95% CI 99%-100%). Stratified by tumor size, *en bloc* resection rates did not show any significant difference. The pooled R0 resection rate was 90% (95% CI 87%-93%). The pooled R0 resection rate was 85% (95% CI, 80%-90%) for large tumor and 92% (95% CI, 87%-93%) for small tumor (*p* < 0.001). Stenosis served as the most common reported complication with pooled incidence of 5% (95% CI 3-8%), followed by perforation (1%, 95% CI 0-1%) and mediastinal emphysema (0% CI 0-1%). The incidence of postoperative stenosis decreased significantly after 2011 (2%, 95% CI 0-3%) compared with that before 2011 (9%, 95% CI 3-8%) (*p* < 0.001).

**Conclusion:**

ESD was an efficient modality for treating superficial esophageal carcinoma, with perfect *en bloc* and R0 resection rate and low complication rate. The most common complication of ESD was stenosis. Although recurrence rate was low, patients should be maintained in a scheduled surveillance program.

## Review

### Introduction

An increasing number of esophageal carcinoma is diagnosed worldwide each year [1]. With the improving of diagnostic technology, esophageal cancer can be diagnosed in early stage. Endoscopic treatment of early esophageal carcinoma has been increasingly conducted around the world, which aims to maintain the integrity of the esophagus and avoid the considerable morbidity and mortality of esophagectomy. Endoscopic mucosal resection (EMR) was the first developed endoscopic resection strategy. However, EMR is sometimes associated with local recurrence, especially when lesions larger than 20 mm are resected in a piecemeal manner [2]. To overcome the limitation of EMR, endoscopic submucosal dissection (ESD) was developed about 10 years ago [3]. ESD allows *en bloc* resection regardless of the size and precise histological assessment of the specimens [4,5]. The best result of ESD is that tumors are excised in one piece with tumor-free lateral basal margins (R0 resection), therefore preventing residual disease and local margins.

Recently, a number of studies were conducted to assess the efficacy and durability of ESD. However, the results of these studies were rather controversial with the R0 resection rate ranging from 71% to 97% [6,7].

Although ESD has been recently recognized as one of the standard treatments for superficial esophageal carcinoma (SEC) in Japan [8], it is highly technique demanding because the wall of the esophagus is thinner than that of stomach and the narrow lumen of the esophagus restricts endoscopic manipulation. As a result, life-threatening complications such as perforation and mediastinal emphysema occur with the incidence of 4-10% [8-10].

We perform this meta-analysis to: (1) analysis the *en bloc* and R0 resection rate of ESD for SEC; (2) analysis the local recurrence rate after ESD; (3) analysis the incidence of complications of ESD to treat SEC.

### Methods

#### Search strategy

This study was conducted following the Meta-analysis Of Observational Studies in Epidemiology guidelines [11]. Electronic literature searches were conducted from 1 January 1980 to 1 December 2013. MEDLINE (PubMed and Ovid), Cochrane Database of Systemic Reviews, Google scholar, and Web of Science were searched for eligible studies. A systemic literature search was performed with the search term “(ESD OR endoscopic submucosal dissection) AND esophag*”. References of all relevant articles were also scanned for potential missing studies. Articles with full text in English were retrieved. The retrieved studies were carefully examined to avoid potential duplicates or overlapping data. No attempt was made to locate unpublished material or contact researchers for unpublished data.

#### Study selection and review process

To be eligible, studies had to meet the following criteria: (1) esophageal cancer was histologically proven; (2) ESD (not EMR) was conducted; (3) *En bloc* or R0 resection rate was reported (4) no age or gender restrictions; (5) published in a peer-reviewed journal from 1 January 1980 to 1 December 2013.

We excluded: (1) non-English language studies; (2) nonhuman studies; (3) reviews and case reports; (4) studies with mean follow up periods less than 6 months; (5) studies with less than 20 patients; (6) study samples that are duplicatly reported.

#### Data collection and quality assessment

Data were collected independently by two investigators (Fenghao Sun and Ping Yuan) from each study using a predefined data table, with disagreements being resolved by consensus. For each study, the following characteristics were collected: first author’s name, year of publication, number of patients, the country in which the study was carried out, study design, faculty, kinds of knife and hooks that was used, ESD and surveillance protocol, *en bloc* and R0 resection rate, time of procedure, occurrence and types of complications, histopathologic types of biopsies and follow up evaluation time. Recorded patient characteristics included age and gender. The quality of each study was assessed using the previously validated Downs and Black [12] instrument, which can assess both randomized and nonrandomized studies. After abstraction, the authors reviewed the evidence tables and discrepancies again were resolved by consensus.

#### Efficacy outcomes and complications

The primary efficacy outcomes were *en bloc* resection, defined as the complete removal of the tumor including the markings into one nonfragmented piece, and R0 resection, defined as complete tumor removal with both lateral and deep margins free of neoplastic cells.

Efficacy outcomes were tabulated according to lesion size (The maximum diameter of the lesion was considered to be the lesion size). The primary durability outcome was local recurrence rate of neoplasm defined histologically after R0 resection. Complications (most commonly perforation, stenosis, mediastinal emphysema and bleeding) were recorded as secondary outcomes. These were ascertained based on the individual study definitions of adverse events. Generally, perforation was diagnosed during ESD. Mediastinal emphysema was diagnosed by the presence of air in the mediastinal space on a chest radiograph or CT scan. Bleeding was defined when patients required blood transfusion during procedure, or a postoperative bleeding that required hemostatic treatment such as endoscopic clipping and coagulation. Stenosis was defined as a stricture that required endoscopic treatment.

#### Statistical analysis

Statistical analysis was performed using Stata version 12.1 (Stata Corp LP, College Station, TX). *En bloc* and R0 resection rates were pooled by either fixed-effects model or random-effects model depending on the heterogeneity across studies. A random-effect model was applied if heterogeneity was significant; otherwise, a fixed-effects model was adopted. I^2^ and Cochran’s Q tests were performed to assess the heterogeneity across studies (for the Cochran Q test, heterogeneity was present if P < 0.05, while values of I^2^ to 25, 50, and 75% represented low, moderate, and high heterogeneity, respectively) [13]. To identify potential sources of heterogeneity, analyses were repeated for each subset of the studies. Additional stratified analysis were performed by sample size (D ≥ 25 mm vs D < 25 mm, 25 mm is the mean diameter of all the lesions of included studies) and year of publication (before 2011 vs after 2011). Although ESD has been improving all the time, there was no turning point where a revolutionary improvement took place. We want to find out whether ESD outcomes have been improved in this decade compared with last decade. All *p* values presented were two-sided. The association was considered significant if the *p* value was less than 0.05.

### Results

#### Search results

**Figure 1:**
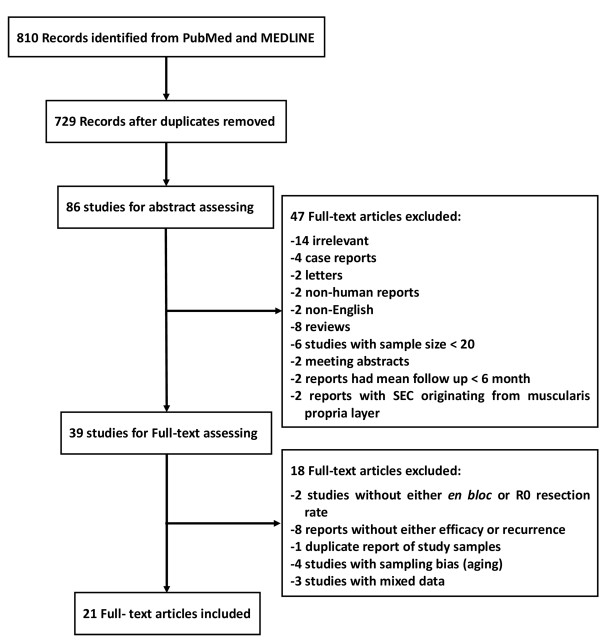
Study flow diagram.

After initial search, 729 articles were identified (Figure 1). Among these, 643 were excluded after the first screening based on abstracts and titles. An additional 65 records were excluded after abstract and full-text review for reasons such as non-English language (n = 2), irrelevant report (n = 14), reviews (n = 8), fewer than 20 subjects (n = 6), mean follow up less than 6 month (n = 2), duplicate reports of study samples (n = 1), meeting abstract (n = 2), no data of either *en bloc* or R0 resection rate (n = 13), SEC originating from the muscularis propria layer (n = 2), Sampling bias (n = 4), combined data (n = 3), animal report (n = 2), case reports and letters (n = 6). Therefore, 21 studies with 1152 patients (1240 lesions) which met the inclusion criteria were included [2,4-10,14-29].

#### Study characteristics

Included studies were published between 2005 and 2013. All but three studies were performed in Japan. The other 3 studies were performed in China, Brazil and Italy, respectively. There were two studies lacking *en bloc* resection rate and one study R0 resection rate. Substantial variability exists in terms of number of patients and instruments. The majority of studies used insulation-tipped knife (IT knife), hook knife, needle knife, or the combination of these knives. Number of patients ranged from 20 to 138. The number of lesions that exceeded 3/4 of esophageal circumference was available only in 6 studies. As for histopathology, 12 studies included squamous cell carcinoma, 8 included adenocarcinoma, and 5 included high-grade dysplasia. Mean follow up period ranged from 12 to 53 months. Four studies did not declare a specific follow-up period but noted to be at least 12 months. Mean ages of patients were around 70 with only one exception. 5 studies did not specify age information. The majority of included patients were male. The characteristics for all included studies were shown in Table 1. All studies were published as full-text articles.

**Table 1:** Characteristics of included studies

**Study**	**Year**	**Country**	**No. patients**	**No. lesion**	**Mean age**	**Mean tumor size (mm)**	**No. of lesions that exceed 3/4 of esophageal circumference**	**Knife**	**Complication stenosis/perforation/mediastinal emphysema**	**Follow up period, mo**	**Local recurrence**	**Quality score**
Arantes. V	2013	Brazil	23	25	68	25	3 (12%)	Flush Knife	0/1/2	21	2	20
Higuchi. K	2013	Japan	52	52	68	20	ND	IT knife/Hook-knife/Needle knife	5/0/0	>12	0	22
Fujinami. H	2013	Japan	35	38	68	31.9	ND	Stag beetle/Hook knife	0/1/8	>12	1	20
Sohara. N	2013	Japan	59	64	68	23	ND	NR	0/1/0	24	0	22
Imai. K	2013	Japan	49	50	72	ND	ND	IT knife	3/0/0	47	0	23
Omae. M	2013	Japan	44	44	70	17	ND	IT knife	0/0/0	33	0	18
Toyonaga. T	2013	Japan	138	138	69	23	ND	IT knife/Hook-knife/Needle knife	0/0/0	53	0	19
Lee. C T	2012	China	20	24	48	33.7	2 (8.3%)	IT knife	3/1/1	12	0	22
Yamashita. T	2011	Japan	71	71	NR	30	11 (15.4%)	IT knife/needle knife	6/1/0	39	0	21
Urabe. Y	2011	Japan	59	79	65	ND	ND	IT knife/Hook knife	4/6/0	36	0	22
Nonaka. K	2010	Japan	25	27	NR	21	ND	Hook knife/Flash knife/flex knife	3/1/0	>12	0	22
Hirasawa. K	2010	Japan	58	58	69	37.7	ND	IT knife/Needle knife	1/0/0	30	0	21
Takahashi. H	2010	Japan	116	116	67	30	26 (22%)	Hook knife/Needle-knife	20/3/0	36	0	23
Repici. A	2010	Italy	20	20	64	32	ND	IT knife/Hook knife	1/0/2	36	0	20
Ishii. N	2010	Japan	35	37	67	22	ND	Hook knife/Flex knife	9/0/0	19	0	20
Ono. S	2009	Japan	84	107	NR	22.9	10 (9.3%)	Flex knife/Splash needle	15/4/1	21	1	22
Fujishiro. M	2009	Japan	79	102	NR	22	9 (8.8%)	Flex-knife/Splash-needle	13/4/0	25	1	16
Ishihara. R	2008	Japan	29	31	64	16	ND	Hook-knife	3/1/0	>12	0	21
Yoshinaga. S	2008	Japan	24	25	62	16.5	ND	IT knife	2/0/0	31	0	21
Kakushima. N	2006	Japan	30	30	70	22.5	ND	Flex knife	0/1/0	15	0	20
Oyama. M	2005	Japan	102	102	NR	28	ND	Hook knife	7/0/6	21	0	17

#### Efficacy outcomes

**Figure 2:**
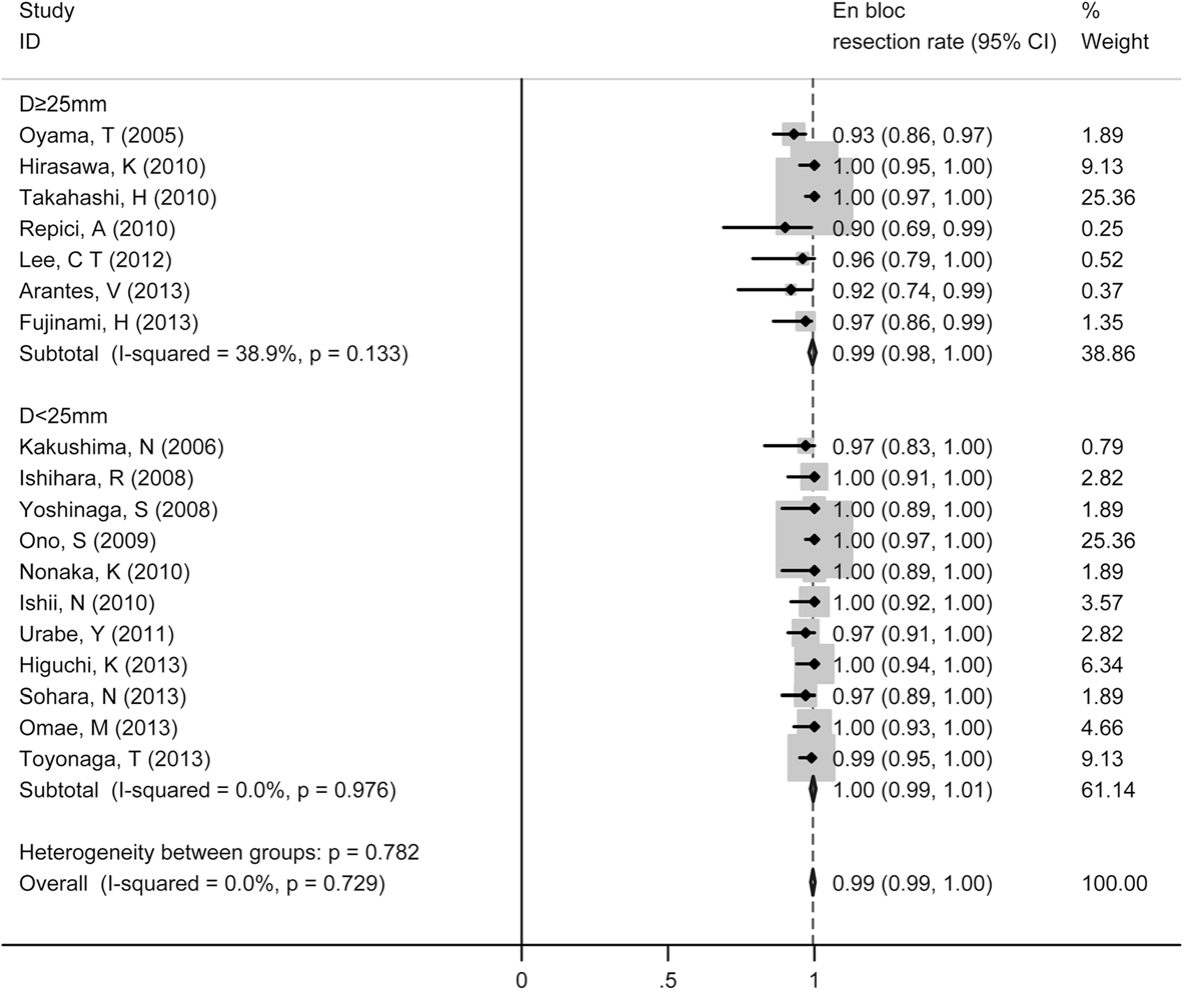
Forest plots of en bloc rate of ESD, stratified by tumor size (D ≥ 25 mm vs. D < 25 mm).

The pooled *en bloc* resection rate was 99% (95% CI 99%-100%). Stratified by tumor size, *en bloc* resection rates did not show any difference. The polled *en bloc* resection rate was 99% (95% CI, 98%-100%) for large tumor (D ≥ 25 mm) and 100% (95% CI, 99%-100%) for small tumor (Figure 2).

**Figure 3:**
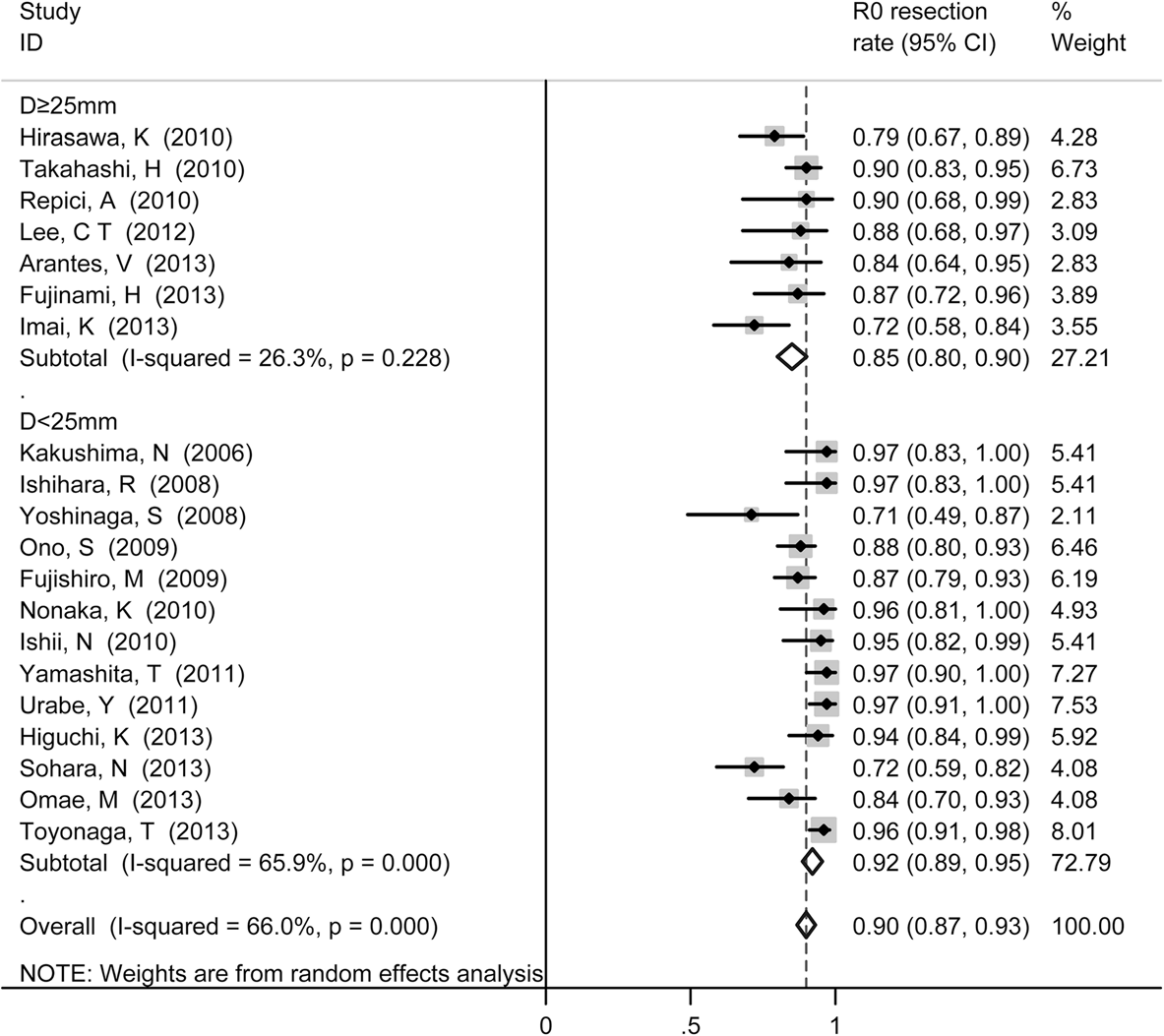
Forest plots of R0 resection rate of ESD, stratified by tumor size (D ≥ 25 mm vs. D < 25 mm).

The pooled R0 resection rate was 90% (95% CI 87%-93%). The pooled R0 resection rate was 85% (95% CI, 80%-90%) for large tumor and 92% (95% CI, 87%-93%) for small tumor (*p* < 0.001) (Figure 3).

#### Complications and recurrence

**Figure 4:**
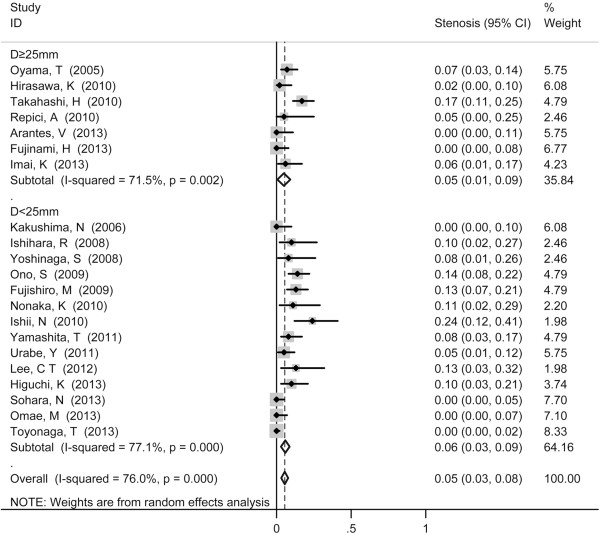
Forest plots of the proportion of patients developed stenosis after ESD, stratified by tumor size (D ≥ 25 mm vs. D < 25 mm).

**Figure 5:**
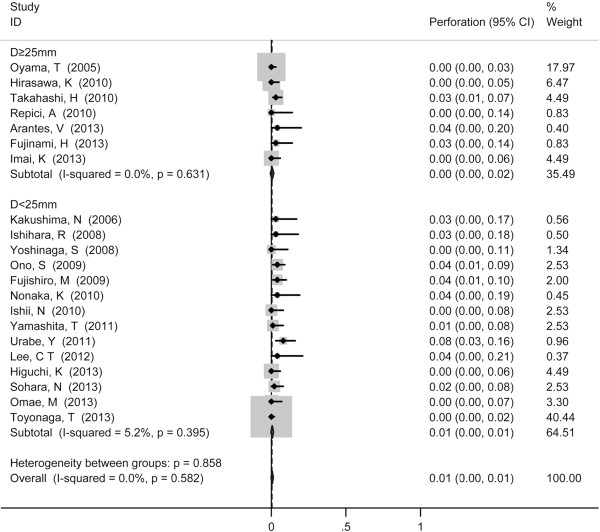
Forest plots of perforation rate during ESD, stratified by tumor size (D ≥ 25 mm vs. D < 25 mm).

**Figure 6:**
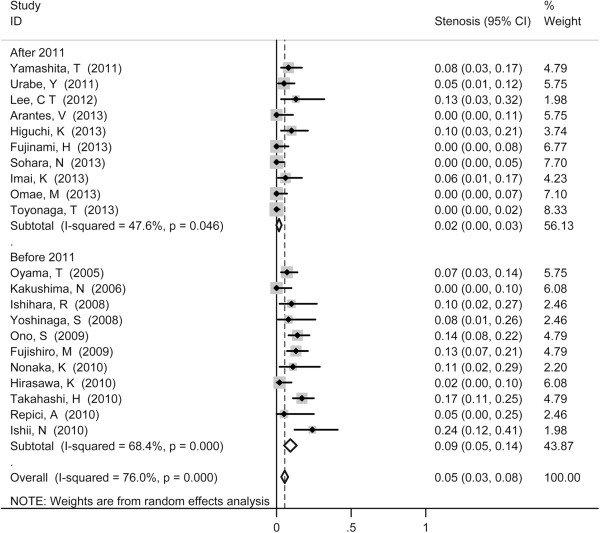
Forest plots of the proportion of patients developed mediastinal emphysema after ESD.

**Figure 7:**
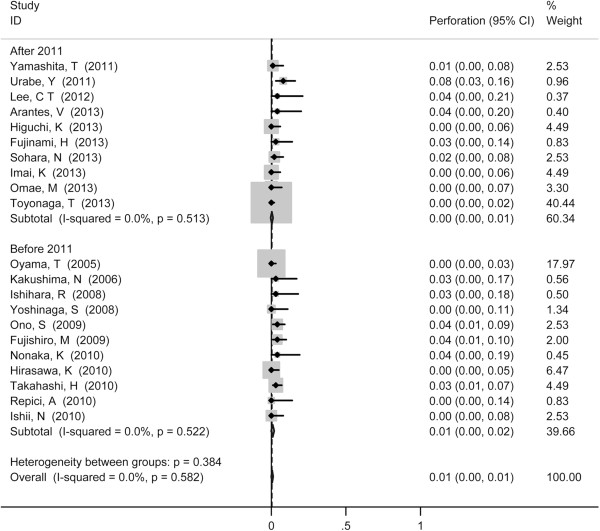
Forest plots of the proportion of patients developed stenosis after ESD, stratified by year of publication (after 2011 vs. before 2011).

**Figure 8:**
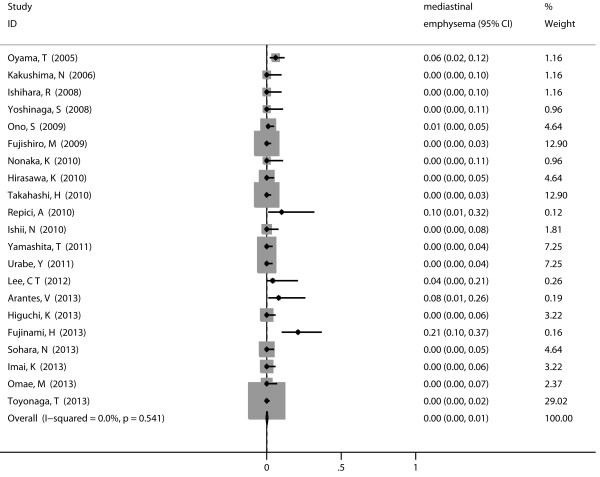
Forest plots of perforation rate during ESD, stratified by year of publication (after 2011 vs. before 2011).

Stenosis served as the most common reported complication with pooled incidence of 5% (95% CI 3-8%) (Figure 4), followed by perforation (1%, 95% CI 0-1%) (Figure 5) and mediastinal emphysema (0%, 95% CI 0-1%) (Figure 6). Tumor size did not show influence on incidence of perforation and stenosis. The incidence of postoperative stenosis decreased significantly after 2011 (2%, 95% CI 0-3%) compared with that before 2011 (9%, 95% CI 3-8%) (*p* < 0.001) (Figure 7). However, this trend was not found in the incidence of perforation (Figure 8). The pooled incidence of mediastinal emphysema was 0% (95% CI 0-1%) (Figure 6). Since mediastinal emphysema occurred only in 20 patients distributed in 6 studies, subgroup analysis would be unnecessary.

Our data showed that only 5 out of 1159 patients developed histopathologically identified local recurrence. 3 of them were managed by an additional ESD and 1 of them underwent EMR and 2 sessions of radiofrequency ablation (RFA) and the other one was not declared.

#### Heterogeneity

Significant heterogeneity was only found in R0 resection rate (I^2^ = 54.6%, *p* < 0.001) and incidence of stenosis (I^2^ = 76.0%, *p* < 0.001).

### Discussion

ESD has been recognized as a reliable treatment for early gastric neoplasms and has been gradually accepted as a preferred method for the endoscopic treatment of SEC [30]. However, the use of ESD for SEC is not well guided. Patients and physician need to be well informed with the risks and benefits of the procedure. In this systematic review and meta-analysis, 1152 patients (1240 lesions) of 21 studies were included. The pooled *en bloc* (99%, 95% CI 99-100%) and R0 (90%, 95% CI 87-93%) resection rates were calculated. We also evaluated the incidence of complication such as perforation (1%, 95% CI 0-1%), stenosis (5%, 95% CI 3-8%) and mediastinal emphysema (0%, 95% CI 0-1%). Local recurrence only occurred in 5 patients. Subgroup analysis was also conducted by tumor size and year of publication. For *en bloc* resection rate, no significant difference was detected between large (D ≥ 25 mm) and small tumor (D < 25 mm). For R0 resection rate, large tumor (85%, 95% CI 80-90%) had a lower R0 resection rate compared with small tumor (92%, 95% CI 89-95%). Most studies reported the *en bloc* resection rate of over 95% except for two. One study reported by Repici et al. had an *en bloc* resection rate of 90%, probably because of the extremely large tumor size (mean = 33 mm) [9]. The other one conducted by Arants et al. reported an *en bloc* resection rate of 92% which might be resulted from its submucosal tunnel dissection procedure instead of circumferential mucosal incision [14]. All but one study achieved R0 resection rate of more than 80%.

ESD for esophageal cancer had been proven to be superior to conventional EMR in terms of the R0 resection rate and the recurrence rate [31]. With ESD, *en bloc* resection can be achieved regardless of the size of the tumor [4]. It was also proved in our study that *en bloc* resection rate did not drop in large tumors. However, R0 resection rate was influenced by the tumor size. Larger tumors were less likely to achieve R0 resection. The reason for such a phenomenon was understandable that resection of larger tumor was more technique-demanding. In this perspective, though ESD can achieve R0 resection in large tumors, the R0 resection rate drops.

The risk of complication is unavoidable since ESD is an endoscopic surgical procedure. Perforation was considered the most common complication during ESD procedures [31]. But our data demonstrated that the perforation rate was only 1% (95% CI, 0-1%), while the incidence of postoperative stenosis was 5% (95% CI, 3-8%), making stenosis the major complication for ESD. A similar result was reported by Sgourakis et al. that the perforation and stenosis rate were 1.8% and 12.2% respectively [32]. Perforation was considered as the most severe complications during ESD procedure. Although it can be temporarily clipped by hemoclips, unstable vital signs during the procedure might result in an urgent operation [3,30]. Sgourakis G et al. reported that esophageal stenosis was statistically more prevalent among patients managed with ESD than EMR [32]. As for mediastinal emphysema, the occurrence of the complication was rare according to the studies included.

Interestingly, significant difference of stenosis occurrence was found before and after the year of 2011, with a trend toward reduced occurrence of stenosis after 2011 (*p* < 0.001). It was reported that a mucosal defect involving more than three-fourths of the esophageal circumference was significantly associated with the development of severe esophageal stricture after EMR [33]. Ono et al. also revealed that circumferential extension and histologic depth of lesion can be reliable independent predictors for postoperative stenosis [34]. However, with only 6 studies specified the number of lesions that exceeded 3/4 of esophageal circumference, it is hard to evaluate the relationship in this study. Instead, we further confirmed in our study that tumor size did not show influence on incidence of perforation and stenosis. The possibility exists that a more rigorous selection of indications may contribute to the decreasing stenosis occurrence. For example, in some centers, lesions that occupied >3/4 esophageal circumference may not suitable for ESD during recent years. Nevertheless, improvements of surgical techniques and devices during last few years should not be ignored. It is demonstrated in Table 1 that the insulation-tipped knife was more widely used in combination of devices after 2011. Before that, hook knife and flex knife were the mainstay devices used in each center. Fujinami et al. reported that the use of the stag beetle (SB) knife for esophageal ESD reduced the risk of complications [16]. Moreover, efficient intervention decreased the incidence and severity of stenosis after endoscopic resection involving more than 75% circumference when preventative dilatation was carried out [31]. Among these interventions, the scheduled endoscopic balloon dilatation (EBD) after ESD and local injection of steroids were the most applied methods. EBD has been a choice in the setting of benign esophageal strictures [35-37]. Local steroid injection into corrosive or anastomotic strictures could achieve remission of dysphagia, with 26.5% to 56% less incidence compared to control groups without intralesional steroid injection [38-40].

It is considered that, compared with EMR, ESD showed a better *en bloc* and R0 resection rate for the treatment of superficial esophageal tumors, leading to a reduced local recurrence rate [31]. Our data of local recurrence showed a similar result that only 5 out of 1159 patients developed histopathologically identified local recurrence. This finding suggested that the possibility of local recurrence should not be overlooked even after R0 resection. Thus, we suggest patients should be maintained in a scheduled surveillance program.

Although stratification by tumor size showed higher R0 resection rate for small tumor (D < 25 mm), heterogeneity was not fully explained. Also, heterogeneity of incidence of stenosis was significant across included studies. It was not surprising owing to the difference in patient samples, settings and protocols, instruments and provider factors. As for stenosis, different center may use different modalities to prevent the occurrence of stenosis, which may explain the significant heterogeneity.

There were also several limitations in this study. First, since most of the included studies were conducted in Japan, it can hardly represent the basic characteristics of patients worldwide. Further efforts should be focused on conducting clinical trials in western centers for further research. Second, heterogeneity was not fully explained. Heterogeneity of R0 resection rate for small tumor and incidence of stenosis were significant. Third, follow-up duration was not long enough which may lead to the underestimation of recurrence rate.

## Conclusions

In summary, this systematic review and meta-analysis showed that (1) ESD was an efficient modality for treating SEC, with perfect *en bloc* and R0 resection rate and low complication rate; (2) Compared with large tumor (D ≥ 25 mm), ESD for small tumor (D < 25 mm) had a higher R0 resection rate; (3) The incidence of stenosis dropped recently compared with several years ago.

## Abbreviations

ESD: Endoscopic submucosal dissection; EMR: Endoscopic mucosal resection; SEC: Superficial esophageal carcinoma; RFA: Radiofrequency ablation; EBD: Endoscopic balloon dilatation; CI: Confidential interval.

## Competing interests

The authors declare that they have no competing interest.

## Authors’ contributions

JH conceived and designed the study. FS analyzed data and wrote the manuscript. PY helped with analysis and data collection. TC helped in data interpretation and date collection. All authors read and approved the final manuscript.

## References

[B1] Parkin DM Bray FI Devesa SS Cancer burden in the year 2000. The global picture Eur J Cancer 2001 9 4 66 10.1016/s0959-8049(01)00267-2 11602373

[B2] Ishihara R Iishi H Uedo N Takeuchi Y Yamamoto S Yamada T Masuda E Higashino K Kato M Narahara H Tatsuta M Comparison of EMR and endoscopic submucosal dissection for en bloc resection of early esophageal cancers in Japan Gastrointest Endosc 2008 9 1066 1072 10.1016/j.gie.2008.03.1114 18620345

[B3] Ono H Kondo H Gotoda T Shirao K Yamaguchi H Saito D Hosokawa K Shimoda T Yoshida S Endoscopic mucosal resection for treatment of early gastric cancer Gut 2001 9 225 229 10.1136/gut.48.2.225 11156645 PMC1728193

[B4] Oyama T Tomori A Hotta K Morita S Kominato K Tanaka M Miyata Y Endoscopic submucosal dissection of early esophageal cancer Clin Gastroenterol Hepatol 2005 9 S67 S70 10.1016/S1542-3565(05)00291-0 16013002

[B5] Fujishiro M Yahagi N Kakushima N Kodashima S Muraki Y Ono S Yamamichi N Tateishi A Shimizu Y Oka M Ogura K Kawabe T Ichinose M Omata M Endoscopic submucosal dissection of esophageal squamous cell neoplasms Clin Gastroenterol Hepatol 2006 9 688 694 10.1016/j.cgh.2006.03.024 16713746

[B6] Yamashita T Zeniya A Ishii H Tsuji T Tsuda S Nakane K Komatsu M Endoscopic mucosal resection using a cap-fitted panendoscope and endoscopic submucosal dissection as optimal endoscopic procedures for superficial esophageal carcinoma Surg Endosc 2011 9 2541 2546 10.1007/s00464-011-1584-6 21359894

[B7] Yoshinaga S Gotoda T Kusano C Oda I Nakamura K Takayanagi R Clinical impact of endoscopic submucosal dissection for superficial adenocarcinoma located at the esophagogastric junction Gastrointest Endosc 2008 9 202 209 10.1016/j.gie.2007.09.054 18226681

[B8] Fujishiro M Kodashima S Goto O Ono S Niimi K Yamamichi N Oka M Ichinose M Omata M Endoscopic submucosal dissection for esophageal squamous cell neoplasms Dig Endosc 2009 9 109 115 10.1111/j.1443-1661.2009.00837.x 19691785

[B9] Repici A Hassan C Carlino A Pagano N Zullo A Rando G Strangio G Romeo F Nicita R Rosati R Malesci A Endoscopic submucosal dissection in patients with early esophageal squamous cell carcinoma: results from a prospective Western series Gastrointest Endosc 2010 9 715 721 10.1016/j.gie.2009.11.020 20363414

[B10] Ono S Fujishiro M Niimi K Goto O Kodashima S Yamamichi N Omata M Long-term outcomes of endoscopic submucosal dissection for superficial esophageal squamous cell neoplasms Gastrointest Endosc 2009 9 860 866 10.1016/j.gie.2009.04.044 19577748

[B11] Stroup DF Berlin JA Morton SC Olkin I Williamson GD Rennie D Moher D Becker BJ Sipe TA Thacker SB Meta-analysis of observational studies in epidemiology J Amer Med Assoc 2000 9 2008 2012 10.1001/jama.283.15.2008 10789670

[B12] Downs SH Black N The feasibility of creating a checklist for the assessment of the methodological quality both of randomised and non-randomised studies of health care interventions J Epidemiol Community Health 1998 9 377 384 10.1136/jech.52.6.377 9764259 PMC1756728

[B13] Higgins J Thompson SG Quantifying heterogeneity in a meta‒analysis Stat Med 2002 9 1539 1558 10.1002/sim.1186 12111919

[B14] Arantes V Albuquerque W Freitas Dias CA Demas Alvares Cabral MM Yamamoto H Standardized endoscopic submucosal tunnel dissection for management of early esophageal tumors (with video) Gastrointest Endosc 2013 9 946 952 10.1016/j.gie.2013.05.031 23810327

[B15] Higuchi K Tanabe S Azuma M Katada C Sasaki T Ishido K Naruke A Katada N Koizumi W A phase II study of endoscopic submucosal dissection for superficial esophageal neoplasms (KDOG 0901) Gastrointest Endosc 2013 9 704 710 10.1016/j.gie.2013.04.182 23680178

[B16] Fujinami H Hosokawa A Ogawa K Nishikawa J Kajiura S Ando T Ueda A Yoshita H Sugiyama T Endoscopic submucosal dissection for superficial esophageal neoplasms using the stag beetle knife Dis Esophagus 2014 9 1 50 54 10.1111/dote.12039 23442212

[B17] Sohara N Hagiwara S Arai R Iizuka H Onozato Y Kakizaki S Can endoscopic submucosal dissection be safely performed in a smaller specialized clinic? World J Gastroenterol 2013 9 528 535 10.3748/wjg.v19.i4.528 23382632 PMC3558577

[B18] Imai K Kakushima N Tanaka M Takizawa K Matsubayashi H Hotta K Yamaguchi Y Ono H Validation of the application of the Japanese curative criteria for superficial adenocarcinoma at the esophagogastric junction treated by endoscopic submucosal dissection: a long-term analysis Surg Endosc 2013 9 2436 2445 10.1007/s00464-012-2755-9 23355156

[B19] Omae M Fujisaki J Horiuchi Y Yoshizawa N Matsuo Y Kubota M Suganuma T Okada K Ishiyama A Hirasawa T Yamamoto Y Tsuchida T Hoshino E Igarashi M Safety, efficacy, and long-term outcomes for endoscopic submucosal dissection of early esophagogastric junction cancer Gastric Cancer 2013 9 147 154 10.1007/s10120-012-0162-5 22692465

[B20] Toyonaga T Man-i M East JE Nishino E Ono W Hirooka T Ueda C Iwata Y Sugiyama T Dozaiku T Hirooka T Fujita T Inokuchi H Azuma T 1,635 Endoscopic submucosal dissection cases in the esophagus, stomach, and colorectum: complication rates and long-term outcomes Surg Endosc 2013 9 1000 1008 10.1007/s00464-012-2555-2 23052530 PMC3572381

[B21] Lee CT Chang CY Tai CM Wang WL Tseng CH Hwang JC Lin JT Endoscopic submucosal dissection for early esophageal neoplasia: a single center experience in South Taiwan J Formos Med Assoc 2012 9 132 139 10.1016/j.jfma.2010.12.002 22423666

[B22] Li QL Zhou PH Yao L Xu MD Zhang YQ Zhong YS Chen WF Submucosal Tumors of the Esophagogastric Junction Originating From the Muscularis Propria Layer: A Large Study of Endoscopic Submucosal Dissection Gastrointest Endosc 2012 9 157 10.1016/j.gie.2012.01.037 22459663

[B23] Shi Q Zhong YS Yao LQ Zhou PH Xu MD Wang P Endoscopic submucosal dissection for treatment of esophageal submucosal tumors originating from the muscularis propria layer Gastrointest Endosc 2011 9 1194 1200 10.1016/j.gie.2011.07.039 21963065

[B24] Urabe Y Hiyama T Tanaka S Yoshihara M Arihiro K Chayama K Advantages of endoscopic submucosal dissection versus endoscopic oblique aspiration mucosectomy for superficial esophageal tumors J Gastroenterol Hepatol 2011 9 275 280 10.1111/j.1440-1746.2010.06503.x 21261716

[B25] Nonaka K Arai S Ishikawa K Nakao M Nakai Y Togawa O Nagata K Shimizu M Sasaki Y Kita H Short term results of endoscopic submucosal dissection in superficial esophageal squamous cell neoplasms World J Gastrointest Endosc 2010 9 69 74 10.4253/wjge.v2.i2.69 21160693 PMC2999061

[B26] Hirasawa K Kokawa A Oka H Yahara S Sasaki T Nozawa A Tanaka K Superficial adenocarcinoma of the esophagogastric junction: long-term results of endoscopic submucosal dissection Gastrointest Endosc 2010 9 960 966 10.1016/j.gie.2010.07.030 21034897

[B27] Takahashi H Arimura Y Masao H Okahara S Tanuma T Kodaira J Kagaya H Shimizu Y Hokari K Tsukagoshi H Shinomura Y Fujita M Endoscopic submucosal dissection is superior to conventional endoscopic resection as a curative treatment for early squamous cell carcinoma of the esophagus (with video) Gastrointest Endosc 2010 9 255–264 261 264 10.1016/j.gie.2010.02.040 20541198

[B28] Ishii N Horiki N Itoh T Uemura M Maruyama M Suzuki S Uchida S Izuka Y Fukuda K Fujita Y Endoscopic submucosal dissection with a combination of small-caliber-tip transparent hood and flex knife is a safe and effective treatment for superficial esophageal neoplasias Surg Endosc 2010 9 335 342 10.1007/s00464-009-0560-x 19517169

[B29] Kakushima N Yahagi N Fujishiro M Kodashima S Nakamura M Omata M Efficacy and safety of endoscopic submucosal dissection for tumors of the esophagogastric junction Endoscopy 2006 9 170 174 10.1055/s-2005-921039 16479425

[B30] Ohkuwa M Hosokawa K Boku N Ohtu A Tajiri H Yoshida S New endoscopic treatment for intramucosal gastric tumors using an insulated-tip diathermic knife Endoscopy 2001 9 221 226 10.1055/s-2001-12805 11293753

[B31] Koike T Nakagawa K Iijima K Shimosegawa T Endoscopic resection (endoscopic submucosal dissection/endoscopic mucosal resection) for superficial Barrett’s esophageal cancer Dig Endosc 2013 9 Suppl 1 20 28 23480400 10.1111/den.12047

[B32] Sgourakis G Gockel I Lang H Endoscopic and surgical resection of T1a/T1b esophageal neoplasms: a systematic review World J Gastroenterol 2013 9 1424 1437 10.3748/wjg.v19.i9.1424 23539431 PMC3602502

[B33] Katada C Muto M Manabe T Boku N Ohtsu A Yoshida S Esophageal stenosis after endoscopic mucosal resection of superficial esophageal lesions Gastrointest Endosc 2003 9 165 169 10.1067/mge.2003.73 12556777

[B34] Ono S Fujishiro M Niimi K Goto O Kodashima S Yamamichi N Omata M Predictors of postoperative stricture after esophageal endoscopic submucosal dissection for superficial squamous cell neoplasms Endoscopy 2009 9 661 665 10.1055/s-0029-1214867 19565442

[B35] Isomoto H Yamaguchi N Nakayama T Hayashi T Nishiyama H Ohnita K Takeshima F Shikuwa S Kohno S Nakao K Management of esophageal stricture after complete circular endoscopic submucosal dissection for superficial esophageal squamous cell carcinoma BMC Gastroenterol 2011 9 46 10.1186/1471-230X-11-46 21542926 PMC3111579

[B36] Isomoto H Yamaguchi N Endoscopic submucosal dissection in the era of proton pump inhibitors J Clin Biochem Nutr 2009 9 205 10.3164/jcbn.SR09-01 19430607 PMC2675020

[B37] Ezoe Y Muto M Horimatsu T Morita S Miyamoto S Mochizuki S Minashi K Yano T Ohtsu A Chiba T Efficacy of preventive endoscopic balloon dilation for esophageal stricture after endoscopic resection J Clin Gastroenterol 2011 9 222 227 10.1097/MCG.0b013e3181f39f4e 20861798

[B38] Yamaguchi N Isomoto H Nakayama T Hayashi T Nishiyama H Ohnita K Takeshima F Shikuwa S Kohno S Nakao K Usefulness of oral prednisolone in the treatment of esophageal stricture after endoscopic submucosal dissection for superficial esophageal squamous cell carcinoma Gastrointest Endosc 2011 9 1115 1121 10.1016/j.gie.2011.02.005 21492854

[B39] Hashimoto S Kobayashi M Takeuchi M Sato Y Narisawa R Aoyagi Y The efficacy of endoscopic triamcinolone injection for the prevention of esophageal stricture after endoscopic submucosal dissection Gastrointest Endosc 2011 9 1389 1393 10.1016/j.gie.2011.07.070 22136782

[B40] Hanaoka N Ishihara R Takeuchi Y Uedo N Higashino K Ohta T Kanzaki H Hanafusa M Nagai K Matsui F Intralesional steroid injection to prevent stricture after endoscopic submucosal dissection for esophageal cancer: a controlled prospective study Endoscopy 2012 9 1007 1011 22930171 10.1055/s-0032-1310107

